# Human platelet lysate: a potential therapeutic for intracerebral hemorrhage

**DOI:** 10.3389/fnins.2024.1517601

**Published:** 2025-01-15

**Authors:** Dachang Qiu, Lin Wang, Lanlan Wang, Yongfei Dong

**Affiliations:** ^1^Wuxi School of Medicine, Jiangnan University, Wuxi, China; ^2^Department of Geriatrics, The First Affiliated Hospital of USTC, Division of Life Sciences and Medicine, University of Science and Technology of China, Hefei, China; ^3^Department of Neurosurgery, The First Affiliated Hospital of USTC, Division of Life Sciences and Medicine, University of Science and Technology of China, Hefei, China

**Keywords:** bioactive factors, treatment, pathophysiology, intracerebral hemorrhage, human platelet lysate

## Abstract

Intracerebral hemorrhage (ICH) is a major public health challenge worldwide, and is associated with elevated rates of mortality, disability, and morbidity, especially in low- and middle-income nations. However, our knowledge of the detailed molecular processes involved in ICH remains insufficient, particularly those involved in the secondary injury stage, resulting in a lack of effective treatments for ICH. Human platelet lysates (HPL) are abundant in bioactive factors, and numerous studies have demonstrated their beneficial effects on neurological diseases, including their anti-neuroinflammatory ability, anti-oxidant effects, maintenance of blood–brain barrier integrity, and promotion of neurogenesis. In this review, we thoroughly explore the potential of HPL for treating ICH from three critical perspectives: the rationale for selecting HPL as a treatment for ICH, the mechanisms through which HPL contributes to ICH management, and the additional measures necessary for HPL as a treatment for ICH. We elucidate the role of platelets in ICH pathophysiology and highlight the limitations of the current treatment options and advancements in preclinical research on the application of HPL in neurological disorders. Furthermore, historical developments and preparation methods of HPL in the field of biomedicine are discussed. Additionally, we summarize the bioactive molecules present in HPL and their potential therapeutic effects in ICH. Finally, we outline the issues that must be addressed regarding utilizing HPL as a treatment modality for ICH.

## Graphical abstract

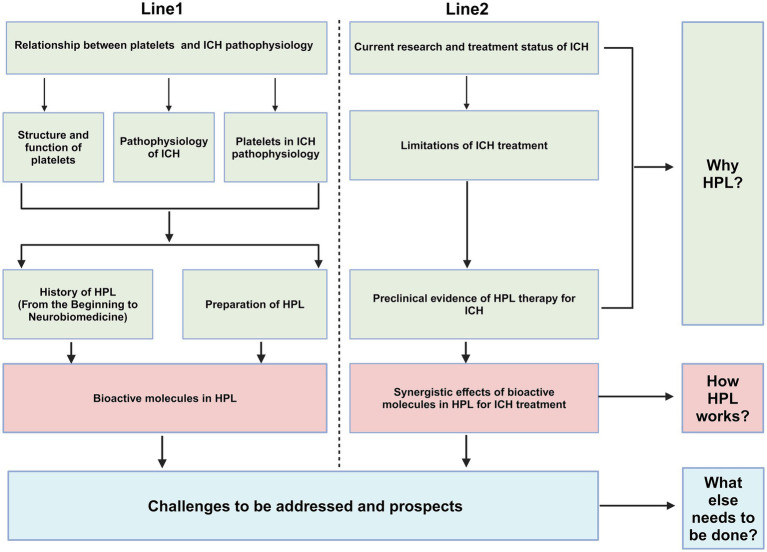

## 1 Introduction

Intracerebral hemorrhage (ICH) is a prevalent cerebrovascular disease associated with high mortality and disability rates. ICH constituted 27.9% of stroke occurrences in 2019 (Collaborators, 2021), and there were 10–30 cases of cerebral hemorrhage per 100,000 individuals globally (Qureshi et al., 2009). In low-income areas, the age-standardized stroke-related mortality rate is 3.6 times higher than that in high-income areas (Collaborators, 2021). Furthermore, from 1990 to 2019, there was a significant increase in the incidence of ICH (Collaborators, 2021); thus, ICH presents a considerable challenge as a serious global public health risk, especially in economically disadvantaged nations. In the past decade, an increasing number of intervention measures have been used to target early acute hematoma in ICH (Al-Kawaz et al., 2020). However, existing treatment for ICH is insufficient. Therefore, recent studies have focused on identifying the precise therapeutic targets and developing more effective alternative therapies for ICH.

HPL is a biomaterial rich in bioactive factors (Schallmoser et al., 2020). In recent years, the beneficial effects of HPL in the treatment of neurological diseases have become increasingly evident (Zhang et al., 2015; Brambilla et al., 2023). Furthermore, owing to the simplicity of HPL production and the ability of HPL to reduce unreasonable platelet waste, it holds great promise as a therapeutic, especially in low-income countries. Research on the use of HPL for the treatment of ICH is currently in the preclinical stage. However, the significant amount of experimental data on HPL in neurological diseases suggests that the transition to clinical trials is iminent (Nebie et al., 2022). This review investigated the relationship between platelet count and ICH pathogenesis. We reviewed the current state of research on the clinical application of ICH, highlighting their limitations, and describe the preclinical evidence supporting the use of HPL in the treatment of ICH. Moreover, we described the history of research on HPL and details of its production process. In addition, we describe the synergistic effects of bioactive molecules in HPL on ICH treatment. Finally, we illustrate the issues that need to be addressed to develop ICH as a therapeutic agent.

## 2 Search strategy

A literature search using the PubMed and Web of Science databases was conducted, with the search scope ending in December 2024. The following keywords were used for both database searches: human platelet lysates, intracerebral hemorrhage, pathophysiology, brain, neuroinflammation, neuronal death, blood–brain barrier, quality, and safety. No filter conditions were applied during the search process.

A search for the keyword “human platelet lysate” in PubMed revealed 1,926 related papers as of November 2024, with the earliest document dating back to 1959. The number of studies on HPL began to significantly increase approximately by 2013, and numerous studies exist on the potential value of HPL in research on various diseases. Searches using the keywords “human platelet lysate” and “brain” revealed 62 relevant papers. The earliest preclinical study exploring the therapeutic potential of HPL in neurological diseases was published by Hayon et al. (2013). Since then, several preclinical studies have been published on the role of the HPL in various brain diseases, including Parkinson’s disease, traumatic brain injury, cerebral infarction, and spinal cord injury (SCI). However, to date, no randomized controlled clinical studies have been conducted. Additionally, when searching across all platforms using the keywords “platelet lysate” and “intracerebral hemorrhage,” we found only one recently published article that described the potential of HPL to treat secondary injuries in ICH (Qiu et al., 2024), indicating that more in-depth research is needed in this area.

## 3 Relationship between platelets and ICH pathophysiology

### 3.1 New perspectives on platelets

Platelets are non-nucleated cells found in the blood and are essential for maintaining effective blood clotting within blood vessels. These cells contain various particles (Iyer and Dayal, 2020), such as alpha, dense, and lysosomal granules, which contain various bioactive molecules, including growth factors, cytokines, and enzymes (Nebie et al., 2022). The platelet surface is equipped with multiple receptors, including the glycoprotein Ib-IX-V and IIb-IIIa complexes. These receptors are essential for facilitating the attachment of platelets to blood vessel walls and promoting platelet clustering. Upon activation, platelets undergo a morphological transformation from a disk shape to an irregular form, accompanied by the release of granular contents, which promotes blood coagulation (Golebiewska and Poole, 2015; Holinstat, 2017). After injury to the blood vessels, platelets quickly attach to the wall of the damaged vessel, creating a plug made of platelets. Subsequently, the coagulation cascade is triggered by the release of various factors by platelets, ultimately creating stable fibrin clots.

Furthermore, platelets are vital for managing anticoagulant and fibrinolytic systems, ensuring proper blood fluidity and preventing excessive clotting (Golebiewska and Poole, 2015). However, recent studies have suggested that platelets could be classified as a novel type of immune cell. Platelets interact with immune cells through various mechanisms and participate in inflammatory responses. Additionally, platelets can release cytokines such as platelet factor 4 (PF4), which regulates immune cell activity (Golebiewska and Poole, 2015; Nicolai et al., 2024). Platelets are activated during inflammation to release bioactive factors that exert pro-inflammatory and anti-inflammatory effects. This bidirectional regulatory capacity allows platelets to assume complex roles in the inflammatory response. Consequently, platelets significantly contribute to many systemic diseases, particularly central nervous system disorders, such as ICH. They are not only involved in maintaining the integrity of the blood–brain barrier (BBB) but may also interact with other immune cells, influencing both inflammatory responses and neuroregeneration processes (Ebermeyer et al., 2021; Mao et al., 2022). It’s worth noting that a clinical study also demonstrated the predictive ability of platelets in the prognosis of ICH (Xu et al., 2024).

### 3.2 ICH pathophysiology

Primary brain injury (PBI) occurs when mechanical damage to blood vessels causes blood to flow into the brain parenchyma, forming a hematoma that compresses the surrounding brain tissue. Mechanical compression leads to ischemia of blood vessels surrounding the hematoma, resulting in swelling of adjacent brain tissue and increased intracranial pressure (ICP) (Peng et al., 2019). This can be fatal if the hematoma compresses the brainstem (Wilkinson et al., 2018). The occurrence of hematoma is a dynamic process, with approximately 30% of patients experiencing hematoma growth after onset (Davis et al., 2006); therefore, preventing hematoma growth is crucial for preventing ICH (Veltkamp and Purrucker, 2017).

**Figure 1:**
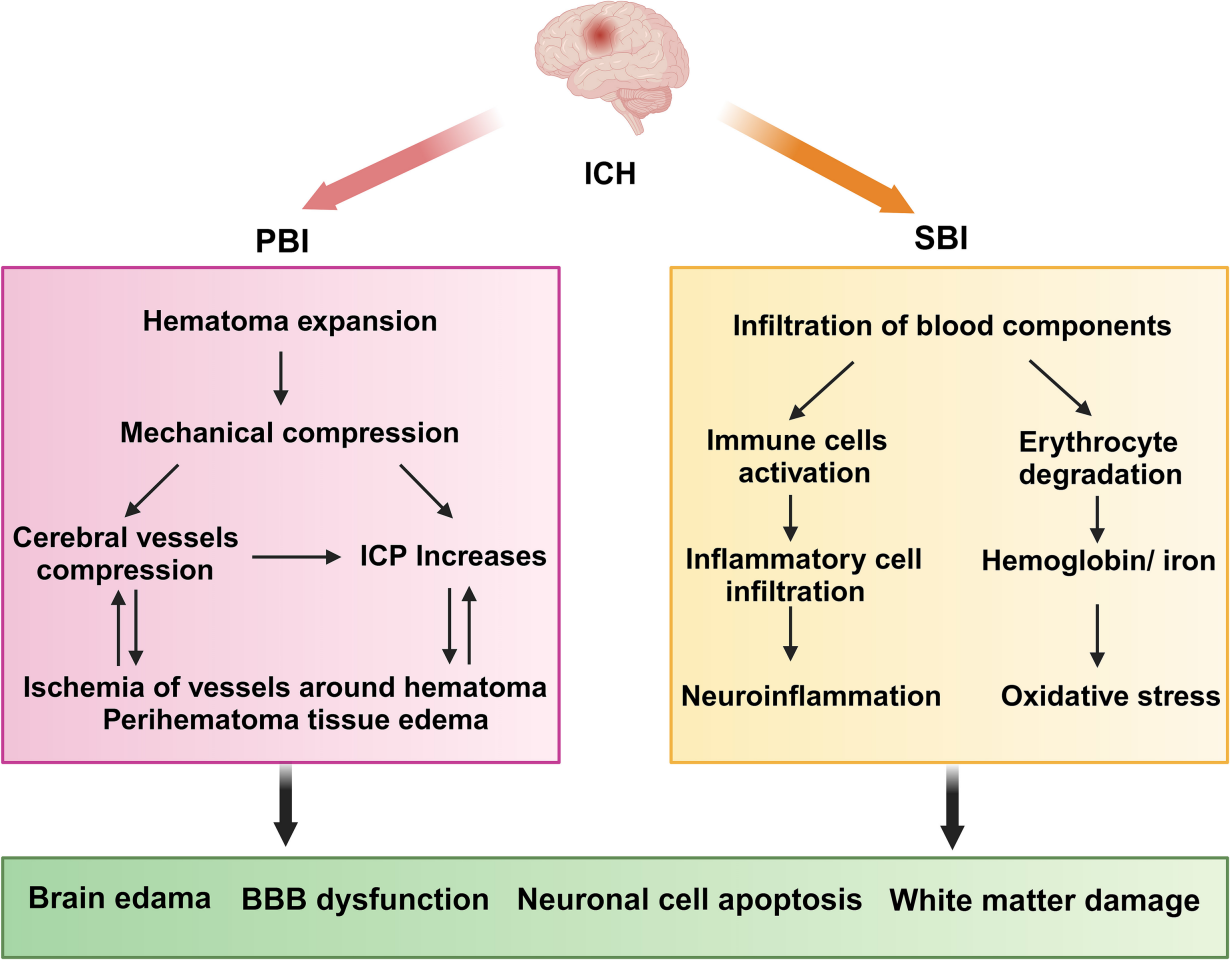
ICH pathophysiology. ICH, Intracerebral hemorrhage; PBI, primary brain injury; SBI, Secondary brain injury; ICP, intracranial pressure; BBB, blood–brain barrier. PBI: The growth of a hematoma causes compression of nearby blood vessels, which leads to ischemia and swelling in the adjacent brain tissue, along with a rise in intracranial pressure. SBI: neutrophils move into the hematoma region while resident immune cells, including microglia, activate and generate pro-inflammatory cytokines. Concurrently, erythrocytes from the hematoma are broken down, releasing hemoglobin and Fe2+. Collectively, these factors contribute to neuroinflammation and oxidative stress, which ultimately result in damage to the blood–brain barrier, exacerbating cerebral hematoma and inducing adjacent edema.

Secondary brain injury (SBI) occurs after primary injury, with microglial activation occurring minutes after ICH. Neutrophils are observed in the hematoma region at 4 h post-ICH and can peak within 3 days. These cells release pro-inflammatory factors, chemokines, and oxidative stress mediators that contribute to neuroinflammation, oxidative stress, and damage to the BBB (Mracsko and Veltkamp, 2014; Chen Y. et al., 2021; Magid-Bernstein et al., 2022). The products of red blood lysis can induce oxidative stress, thereby exacerbating SBI. These products include hemoglobin and Fe2+ (Figure 1) (Keep et al., 2014). Administering lysed red blood cells into the cerebral ventricles of rats results in quicker and more pronounced brain edema compared with the effects of administering concentrated red blood cells (Xi et al., 1998). Furthermore, administration of iron chelators can mitigate brain damage in animal models (Gu et al., 2009; Okauchi et al., 2010). Thrombin plays a crucial role in the development of SBI. While high concentrations of thrombin can lead to nerve damage in laboratory settings (Donovan et al., 1997), low concentrations possess neuroprotective properties (Vaughan et al., 1995). In addition, thrombin has been found to have the potential to promote neurogenesis (Yang et al., 2008).

### 3.3 Role of platelets in ICH pathophysiology

Previous reports have described the structural similarities between platelets and neuronal cells (Canobbio, 2019). The alpha granules in platelets, which store bioactive molecules, are analogous to the large dense-core vesicles found in neurons (Burnouf and Walker, 2022); the process through which platelets transmit signals bears a subtle resemblance to the transmission of signals at neuronal terminal synapses. In both cases, exocytosis is triggered by Ca2^+^ (Reed et al., 2000; Burnouf and Walker, 2022). Dense platelet granules are rich in neurotransmitters such as epinephrine, dopamine, glutamate, and gamma-aminobutyric acid (GABA) (Kaneez and Saeed, 2009; Burnouf and Walker, 2022).

**Figure 2:**
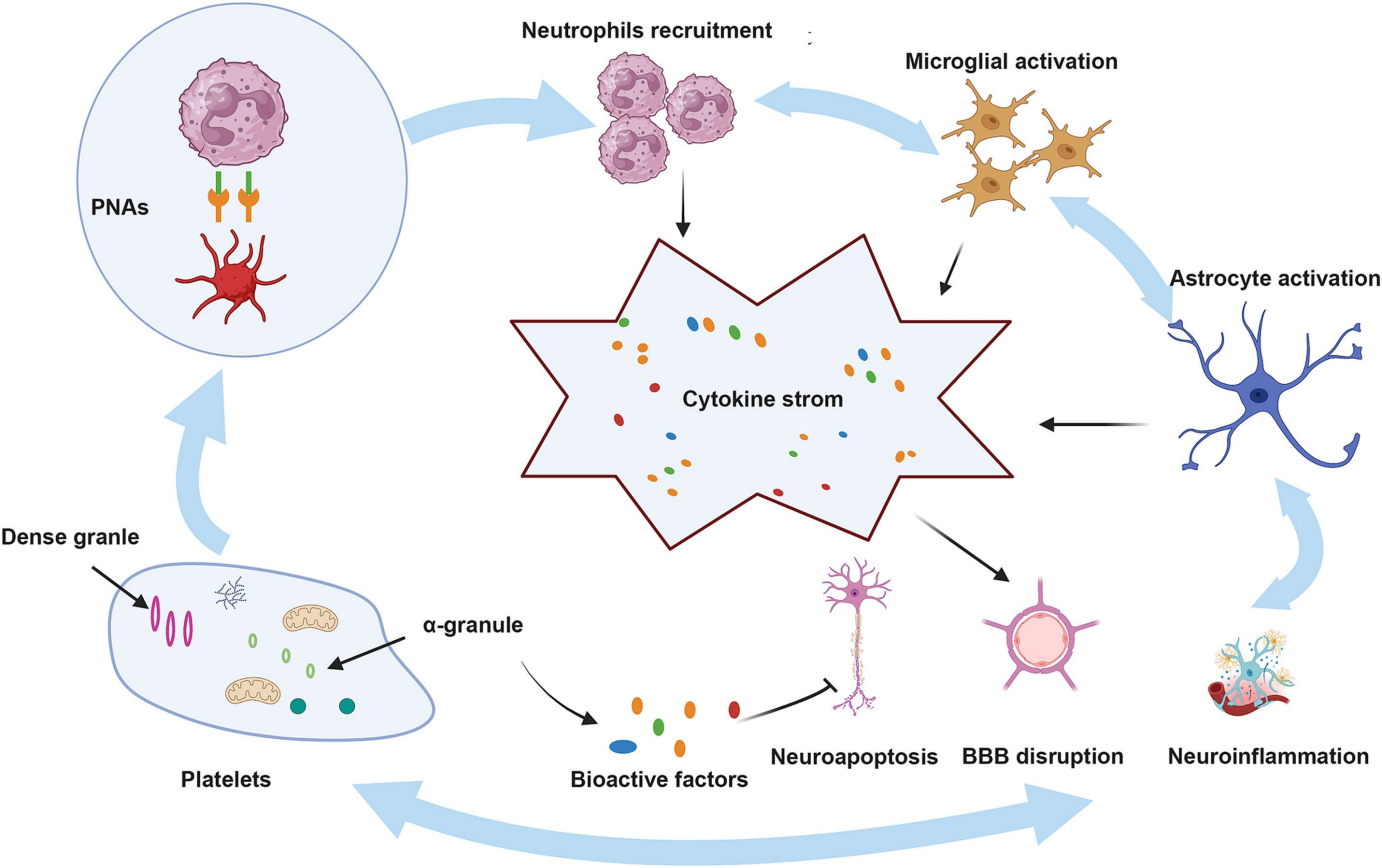
The role of platelets in ICH. BBB, blood–brain barrier; PNAs, platelet–neutrophil aggregates. Upon injury, platelets that have been activated attach to neutrophils through PSGL-1 and CD40, utilizing the surface receptors CD62P and CD40L, which leads to the development of PNAs. These aggregates enhance neutrophil function by releasing inflammatory mediators and cytokines. Moreover, neutrophils that are recruited, in conjunction with activated microglia and astrocytes, produce a range of pro-inflammatory substances, resulting in a cytokine storm that intensifies ICH. Conversely, bioactive factors present in the alpha granules of platelets may mitigate these pathological processes by reducing neuroinflammation, neuroapoptosis, and damage to the BBB.

Platelets play a crucial role in neuroimmune responses. They bind to the corresponding ligands on neutrophils via receptors on their surfaces, which promote the activation and aggregation of neutrophils, ultimately contributing to BBB damage and exacerbating neurological injury, as shown in detail in Figure 2 (Chou et al., 2023). A previous review highlighted an innovative function of platelets in immune responses, emphasizing their role in the activation, differentiation, and suppression of T cells such as Th1, Th17, and Treg cells. Thus, studying the relationship between platelets and T cells could serve as a paradigm for understanding the interaction between the immune and nervous systems (Ponomarev, 2018). Although the immune response serves as a defense mechanism, it can also damage the body. This protective effect is associated with bioactive factors that are stored in the alpha granules of platelets and are closely linked to the regeneration and repair processes. Recently, these bioactive factors have demonstrated positive effects in various animal disease models, including those of diseases involving the nervous system (Griffith et al., 2021; Nebie et al., 2021b; Nebie et al., 2022; Alhawari et al., 2023; Grzelak et al., 2024). These factors can enhance the neurotrophic properties of adipose stem cells to a greater extent than differentiated Schwann cells (Brambilla et al., 2023), improve wound healing, promote neuronal differentiation *in vitro*, and promote angiogenesis and neurogenesis, while exerting neuroprotective effects in rats following stroke (Hayon et al., 2013; Nebie et al., 2021a). Although several of the above-mentioned studies have revealed positive effects of the bioactive factors on neuroimmunity, the potential role of the HPL in ICH pathogenesis has not yet been elucidated. Further research in this area is required to address this gap.

## 4 Progress in ICH treatment research and its limitations

### 4.1 Surgery

Individuals experiencing severe ICH generally receive urgent surgical intervention shortly following the occurrence of the injury. Surgical procedures may involve external ventricular drainage (EVD), craniotomy to remove hematomas, and less invasive surgical methods. Notably, over 40% of those with ICH are likely to experience intraventricular hemorrhage (IVH) (Hallevi et al., 2008). However, Infections caused by EVD are frequently fatal in neurologically critical patients, and the high incidence of this complication warrants significant attention (Zhou et al., 2023). Increased intracranial pressure resulting from the obstruction of cerebrospinal fluid flow due to IVH necessitates urgent EVD. In certain cases, combined thrombolysis may also be required. While craniotomy for hematoma extraction is the most thoroughly researched surgical technique at present, it continues to be a subject of debate. The earliest experimental findings indicate that craniotomy offers no substantial advantage regarding mortality rates (McKissock et al., 1961). Although prompt surgical intervention can critically preserve patients’ lives, existing studies do not show a considerable advantage of early surgery concerning long-term mortality and disability. This is mainly attributed to the risk of surgical complications, such as bleeding and infections (Mendelow et al., 2013). As a result, in craniotomy procedures requiring the extraction of substantial bone flaps and the exposure of broad sections of brain tissue, different surgical approaches have been established, such as minimally invasive techniques. In contrast to open surgery, minimally invasive methods can significantly lower the risks linked to hematoma extraction and surgical trauma. In 1989, Dr. Auer carried out the initial controlled study on minimally invasive surgery, showing that this method is beneficial for mortality rates as well as overall patient results (Auer et al., 1989), however, these findings were restricted to patients with subcortical hemorrhage. Recent studies have indicated that minimally invasive surgery does not provide advantages in long-term functional outcomes when compared to conservative treatments (de Oliveira Manoel, 2020).

### 4.2 Blood pressure control and reversal antiplatelet drugs

Blood pressure is acknowledged as a crucial element in the worsening of acute ICH as the hematoma enlarges. An examination of findings from two extensive randomized controlled trials suggests that higher early variability in blood pressure correlates with less favorable functional outcomes (Chung et al., 2018; Divani et al., 2019; Moullaali et al., 2019). However, results from the large randomized controlled trial of the INTERACT2 study indicated that in patients with ICH, intensive blood pressure lowering did not significantly reduce the primary outcomes of mortality or severe disability (Anderson et al., 2013). The reversal of antiplatelet therapy is an essential element in cases of treatment for ICH. Currently, ICH related to antiplatelets represents a considerable challenge in medical treatment. A frequently utilized approach for reversing antiplatelet therapy is platelet transfusion; nonetheless, this procedure may worsen thrombotic events. As of now, there are no dedicated medications specifically designed to counteract the impacts of antiplatelet therapy. While not commonly used in medical practice, idarucizumab has demonstrated efficacy in reversing anticoagulant actions in a major study involving individuals with gastrointestinal bleeding (Pollack et al., 2017). Multiple studies have shown that andexanet-*α* exhibits significant hemostatic effects (Connolly et al., 2016; Connolly et al., 2019). However, approximately 10% of all trial subjects experienced thrombotic events (Siegal et al., 2015). A significant randomized study involving the antifibrinolytic medication tranexamic acid (TXA) showed a decrease in early mortality rates among individuals with ICH treated with TXA. Nevertheless, it did not indicate a meaningful effect on functional outcomes at 90 days or on overall mortality rates (Sprigg et al., 2018).

### 4.3 Targeting neuroinflammation

Investigating critical components of the inflammatory pathway is presently regarded as an encouraging therapeutic approach to suppress neuroinflammation after ICH. The activation of microglia and macrophages is vital in numerous inflammatory processes, playing a key part in the SBI and acting as a major source of chemokines and inflammatory factors (Gang et al., 2018), Modulating microglia and macrophages to decrease the ratio of M1 /M2 polarized cells is currently a prominent focus in the research and treatment of ICH. In mice with ICH, it has been demonstrated that Irisin reduces M1 macrophage levels while promoting an increase in M2 macrophages, thus exhibiting an anti-neuroinflammatory effect. Furthermore, a different preclinical investigation revealed that a Dectin-1 inhibitor can facilitate the conversion of M1 macrophages to M2 macrophages (Fu et al., 2021; Wang et al., 2022). While many research efforts have illustrated the capability of these agents to suppress neuroinflammation, most have not advanced to the stage of clinical trials. Earlier studies indicated that antagonists of TNF-*α* can decrease PHE and improve neurological results (King et al., 2013). Nevertheless, there is a significant lack of clinical studies examining the effectiveness of TNF-α antagonists for addressing ICH.

### 4.4 Targeting oxidative stress

Currently, there are no specific therapies targeting oxidative stress that are widely implemented in the clinical treatment of ICH. After ICH occurs, the infiltration of blood and the breakdown of red blood cells are major contributors to oxidative stress injury and are intricately linked to ferroptosis. An earlier investigation demonstrates that iron chelators can alleviate the detrimental impacts of hemoglobin and Fe2+ in relation to ICH (Wu et al., 2012). Ceruloplasmin (CP) has been shown to reduce brain injury by promoting the transformation of Fe2+ into Fe3+ (Chen et al., 2022). GPX has emerged as a central inhibitory factor of ferroptosis in recent years, capable of reducing oxidative stress by inhibiting ferroptosis. Therefore, GPX mimics is a promising strategy for mitigating oxidative stress. Ebselen, a well-known GPX mimic, has demonstrated its antioxidant potential in several clinical trials (Yamaguchi et al., 1998; Ogawa et al., 1999). Employing Nrf2 activators is also seen as a method to boost antioxidant capacity and reduce damage caused by oxidative stress after ICH. Recombinant C1q/TNF-related protein 9 (rCTRP9) has the ability to stimulate the AdipoR1/APPL1/AMPK/Nrf2 signaling pathway, which helps decrease neurological impairment in a mouse model of ICH (Zhao et al., 2021). The research shows that albumin reduces oxidative stress injury in a rat model of cerebral hemorrhage by activating the ERK/Nrf2/HO-1 signaling pathway (Deng et al., 2021).

### 4.5 Targeted peripheral hematoma edema

At present, no formal guidelines exist for the management and treatment of peripheral hematoma edema (PHE). Several factors limit research on treatment approaches, such as the expansion of hematomas and the different techniques employed to assess PHE (Al-Kawaz et al., 2020). Hypertonic treatments, including mannitol and hypertonic saline, are extensively employed in clinical settings. Although the short-term effects of these therapies have been confirmed, their impact on long-term neurological results is still unclear (Wang et al., 2015). Studies have shown that sulfonylureas can reduce PHE after ICH (Irvine et al., 2019). Additionally, there is a study investigating the role of glyburide in the treatment of PHE in ICH (GATE-ICH, NCT03741530).

The intricacies of ICH pathophysiology are multifaceted, and we still do not fully comprehend certain underlying mechanisms. This leads to considerable difficulties in the treatment of ICH. Surgical methods have not demonstrated improvements in long-term results, and pharmacological therapies lack specificity. Consequently, the available treatment options for ICH are restricted.

In comparison to traditional therapies, (1) HPL could enhance nerve regeneration. Research indicates that HPL has the ability to stimulate the growth of endogenous neural stem cells, aiding in nerve regeneration and repair (Nebie et al., 2019). Conversely, conventional treatments typically prioritize symptom management rather than actively facilitating nerve regeneration. (2) Enhance cognitive abilities. HPL might positively influence cognitive function, representing a significant benefit during recovery following intracerebral hemorrhage (Nebie et al., 2021b). (3) Being a biological agent, HPL could present a reduced likelihood of adverse effects. Traditional therapies, particularly surgical approaches, often involve a higher potential for complications, including infection or postoperative hemorrhage. (4) HPL is easy to utilize. The process of preparing and administering HPL is generally straightforward and applicable in various contexts, whereas traditional treatments frequently necessitate more sophisticated medical infrastructure and expertise. (5) Reduced economic burden. While there may be some costs associated with the preparation and administration of HPL, it might offer a more economical alternative when compared to high-risk surgical procedures and prolonged medication regimens (Nebie et al., 2022). Considering the constraints of conventional treatment approaches for ICH, an increasing number of investigations are exploring innovative methods for its management, leading to significant advancements. Dr. Xu and his team developed a novel hydrogel substance to address ICH, reporting a strategy that integrates neuroprotection with the stimulation of intrinsic nerve regeneration, showcasing considerable therapeutic promise (Wang Y. et al., 2024; Zhang et al., 2024; Xu et al., 2025). Notably, HPL has also indirectly shown neuroprotective and neuroregenerative properties across various studies.

## 5 Historical highlights and preparation of HPL

### 5.1 History of HPL in biomedicine

HPL is currently defined internationally as a biological material rich in protein and growth factors and free of cells (Schallmoser et al., 2020). The development of HPL is strongly connected to research on platelet-rich plasma (PRP), especially because of its significant cytokines content. In the 1980s and 1990s, researchers identified multiple growth factors derived from platelets and explored their possible functions in healing wounds and regenerating tissue (Lynch et al., 1987; Pierce et al., 1989; Pierce et al., 1991; Hosgood, 1993). In 1983, Cowan employed the freeze–thaw method to prepare PL to investigate its effects on tumors (Cowan et al., 1983). In 2005, Doucet was the first to utilize HPL as a substitute for fetal bovine serum (FBS) to promote mesenchymal stem cell growth, revealing its significant potential for stem cell cultivation (Doucet et al., 2005). This discovery is of great significance, as it indicates that cell therapy applied to humans can eliminate the need for animal-derived biological materials in culture. This advancement reduces the risk of transmission of zoonotic infections and minimizes immunogenicity. Nowadays, HPL is employed as a substitute for FBS in the media used for cell cultures across different cell types, reducing the likelihood of immune reactions and infections linked to biological materials derived from bovine sources (Burnouf et al., 2016; Schallmoser et al., 2020; Oeller et al., 2021). Investigations into utilizing HPL as an alternative to FBS in the cultivation of different stem cells for treating clinical diseases are consistently on the rise. A systematic review by Palombella demonstrated that, compared to FBS, HPL can enhance the proliferation of adipose stem cells (ASCs) and bone marrow stem cells (BMSCs) by increasing the doubling rate and reducing the doubling time (Palombella et al., 2022). The study by Ballesteros further investigated the effects of varying concentrations of HPL on the proliferation and phenotypic marker expression of ASCs. The findings indicated that higher concentrations of HPL can enhance adipogenesis and osteogenesis while concurrently reducing the expression of the chemotactic factor receptors CXCR2 and CXCR3 (Ballesteros et al., 2020). This shows that by adjusting the HPL concentration, stem cells’ biological characteristics can be regulated to a certain extent, providing the possibility of directional differentiation of stem cells. Based on the application of HPL in cell therapy, neuroscientists have increasingly focused on neurological diseases. Recent studies have demonstrated that Schwann cells differentiated from adipose stem cells (SC-ASCs) can produce abundant neurotrophic factors, indicating their potential utility in the treatment of peripheral nerve injury (PNI). However, challenges remain in the clinical translation of this cell therapy. Brambilla found that ASCs cultured with HPL produce a greater quantity of neurotrophic factors than SC-ASCs cultured under the same conditions. This solves the problems in the clinical translation process of SC-ASCs cell therapy (Brambilla et al., 2023). Other similar studies have demonstrated the significant potential of HPL in innovative cell therapies for the treatment of neurological diseases (Tan et al., 2016; Palombella et al., 2020). The aforementioned studies illustrate the capacity of HPL to address neurological diseases through cell therapy indirectly.

### 5.2 Preparation of HPL

HPL is mainly extracted from platelet concentrate (PC). The platelet content of PC is four to five times that of circulating blood (Schallmoser et al., 2020). Although PC is not difficult to obtain, the preproduction of raw materials for HPL must meet strict standards. (1) The collection, testing, storage, and transportation of raw plasma should comply with regulations, and the quality and legality of the source of raw plasma must be ensured. Raw plasma may contain pathogens associated with blood-borne diseases, such as HIV, HBV, and HCV (Burnouf et al., 2019). (2) Production facilities must obtain appropriate licensing permits (Schallmoser et al., 2020). (3) The production process must be strictly controlled, particularly the removal and/or inactivation of viruses (Bieback et al., 2019). (4) A tracking system must be established from the source of blood collection to the end of production to enable possible problems to be traced (Strunk et al., 2018b).

Moreover, as the starting material for the production of HPL, PC should have the following qualities (Schallmoser et al., 2020): (1) test negative for infectious diseases, viruses, bacteria, and fungi; (2) pH > 6.4 at the end of the shelf life; (3) at least 0.6 × 10^11^ platelets per 40 mL; and (4) a number of platelets per unit >2 × 10^11^ and a number of white blood cells <1 × 10^6^.

Different methods of obtaining PCs may affect the variability of HPL fractions, and several previous studies have reported a reduction in the variability of bioactive factors in PCs from different sources by pooling PCs (Horn et al., 2010; Lohmann et al., 2012). Currently, there is no consensus on the standard size of PCs produced by the Good Manufacturing Practice (GMP) HPL production method, which has been adopted worldwide, with reported sizes ranging from 4 to 125 PC units (Strunk et al., 2018a). Some publications use 10–15 PC units, which is equivalent to the amount of plasma donated by 40–50 people (Schallmoser et al., 2007; Bieback et al., 2009). Thus, 10–15 PC units is the best quantity to reduce variability. However, according to the recommendations of the European Pharmacopeia, the number of PCs should be limited to reduce the risk of infection (Schallmoser et al., 2020).

HPL production typically involves physical and biochemical methods. The most prevalent technique is the freeze–thaw method (74%), followed by the use of biochemical agents to activate platelets (13%), ultrasound (8%), and solvent detergent (2%) (Schallmoser et al., 2020). The freeze–thaw method involves placing platelet-rich plasma at −80°C overnight, then incubating it at 37°C, repeating the process three times, and obtaining a protein-rich solution through repeated centrifugation and filtration (Astori et al., 2016). Ultrasound at a frequency of 20 kHz can also be used to prepare HPL. After 30 min of ultrasonic lysis, 74% of PDGF-AB is released from the platelet granules (Bernardi et al., 2013). Table 1 summarizes the details of the various preparation methods for HPL. The preparation process for HPL is simple, and the primary raw materials required are easy to obtain. This can also prevent PC waste owing to its short shelf life. As a low-cost blood product, HPL holds great medical value. However, strict and unified standards for acquiring and producing raw materials must be established.

**Table 1:** Details of different production methods of HPL.

Method	Detail of product
Freeze and thaw	1. Centrifugation (750–3,000 × g 5–30 min 22°C) and discard supernatant; 2. Resuspended in PBS; 3. Freeze–Thaw for 3–5 cycles; 4. Centrifugation and supernatant recovered; 5. Heated in 56°C for 30 min; 6. Centrifugation and discard precipitate
Freeze and thaw	1. Freeze–Thaw for 3–5 cycles to stimulate platelets lysis; 2. Centrifugation (4,000–6,000 × g 30 min 22°C); 3. Cell debris removal; 4. Supernatant recovered
Ultrasound	1.20 HZ Ultrasound stimulate platelets lysis; 2. Centrifugation (1,600 × g 15 min 22°C); 3. Supernatant recovered
Activation of platelet	1. Platelets activated with thrombin or Cacl_2_/Glass beads; 2. Centrifugation (1,000–6,000 × g 20–30 min 22°C); 3. Discard clot and recover supernatant
Solvent/detergent	1. Solvent detergent (lytic platelets and inactivated virus); 2. Centrifugation (1,000–6,000 × g 15 min 22°C); 3. Cell debris removal; 4. Supernatant recovered

Adapt from Shih and Burnouf (2015) and Nebie et al. (2022).

### 5.3 Therapeutic potential of bioactive factors in HPL

Detected by Elisa and multiplex assays, HPL is abundant in bioactive factors, such as growth factors like brain-derived neurotrophic factor (BDNF), platelet-derived growth factor (PDGF), epidermal growth factor (EGF), vascular endothelial growth factor (VEGF), transforming growth factor-*β* (TGF-β), insulin-like growth factor (IGF), insulin-like growth factor-1 (IGF-1), fibroblast growth factor (FGF), angiopoietin-1, and IGF-binding protein 3 (IGF-BP3) (Astori et al., 2016; Viau et al., 2019; Oeller et al., 2021). Additionally, HPL also contains various chemokines, interleukins, and antioxidants (Nebie et al., 2022). Such as platelet factor 4 (PF4), CC-chemokine ligand 3 (CCL3), macrophage inflammatory protein (MIP-1), macrophage migration inhibitory factor (MIF), interleukin-4 (IL-4), interleukin-10 (IL-10), catalase (CAT), superoxide dismutase (SOD), and glutathione peroxidase (GPX), these bioactive factors not only aid in wound repair and vascular regeneration but also contribute to combating neuroinflammation, oxidative stress, and reducing cell apoptosis (Hassamal, 2023). Table 2 summarizes the bioactive factors present in HPL and their active roles in ICH based on previously reported platelet components (Viau et al., 2019).

**Table 2:** Bioactive factors contained in HPL and their potential therapeutic effects in ICH.

Bioactive factor	Potential therapeutic effects on ICH	References
BDNF	Contributes to post-ICH neurogenesis; Treatment of neuronal loss and neurocognitive impairment; Reduces inflammation, apoptosis and astrogliosis	Ko et al. (2018), Ahn et al. (2021), Lin et al. (2023)
IGF	Protection of blood–brain barrier; Improved hippocampus volume and number of neurons after ICH; Reduces inflammation in ICH	Nowrangi et al. (2019), Sun et al. (2020), Vafaee et al. (2020)
EGF	Enhances the survival, growth, and development of OPCs; Protection of the blood–brain barrier	Aguirre et al. (2005), Scafidi et al. (2014), Vinukonda et al. (2016), Yang S. et al. (2019)
MANF	Inhibits neuronal apoptosis through the activation of the Akt/MDM2/p53 signaling pathway in ICH; Reduces mitochondrial dysfunction caused by ROS accumulation; Reduces the apoptotic effect of TNF-αon SH-SY5Y	Zhang et al. (2017), Xu et al. (2018), Zhang et al. (2023)
PDGF	Protection of the blood–brain barrier; Regulates NSC proliferation, migration, differentiation and survival	Erlandsson et al. (2001), Obermeier et al. (2013)
TGF-β	Promotes neurogenesis and reduces apoptosis	Buisson et al. (2003), Ma et al. (2008)
VEGF	Reduces brain edema, neuronal death, and neurological deficits; Promotes endothelial cell proliferation, migration and angiogenesis	Chu et al. (2013), Guo et al. (2017)
GSN	Inhibits apoptosis; neuroprotective effect	Ohtsu et al. (1997), Endres et al. (1999)
FGF	Protection of the blood–brain barrier; Inhibits neuroinflammation by promoting mitophagy and inhibiting the cGAS-STING pathway	Huang et al. (2012), Ma et al. (2024)
GPX	Reduces oxidative damage to mammalian cells	Bhowmick et al. (2015)
SOD	Protective effect of dopaminergic neurodegeneration	Wang et al. (2004)
CAT	Protective effect of dopaminergic neurodegeneration	Wang et al. (2004)
IL-10	Inhibits neuroinflammation and apoptosis; Accelerates hematoma clearance in ICH; Alleviates depression-like behavior and cognitive dysfunction; Reduces ROS accumulation and against OPC loss and white matter injury	Song et al. (2019), Zhang et al. (2020), Li et al. (2021), Han et al. (2023), Wu W. et al. (2024)
IL-4	Promotes hematoma resolution and improved long-term functional recovery in ICH; Promotes neuro-functional recovery and Inhibits neuroinflammation by regulating M1/M2 phenotype microglia polarization	Yang et al. (2016), He et al. (2020), Xu et al. (2020)
MiR-126-3p	Protection of the blood–brain barrier; Reduces brain edema and neuronal damage	Xi et al. (2017), Fu et al. (2019)
PF4	Enhances neurogenesis	Leiter et al. (2019)

Adapt from Nebie et al. (2022); HPL, Human Platelet Lysate; ICH, intracerebral hemorrhage; OPCs, oligodendrocyte progenitor cells; PDGF, platelet-derived growth factor; BDNF, brain-derived neurotrophic factor; TGF-β, transforming growth factor β; EGF, epidermal growth factor; FGF, fbroblast growth factor; VEGF, vascular endothelial growth factor; GSN, gelsolin; IGF, insulin-like growth factor; MANF, mesencephalic astrocyte-derived neurotrophic factor; GPX, glutathione peroxidase; CAT, catalase; ROS, reactive oxygen species; TNF-α, tumor necrosis factor-α; IL-10, interleukin-10; IL-4, interleukin-4; MiR-126-3p, MicroRNAs-126-3p.

Several key factors among these bioactive components, such as BDNF, TGF-*β*, and IL-10, have been the subject of extensive investigation concerning their involvement in ICH. Notably, BDNF is widely acknowledged as a neurotrophic factor. Earlier studies have shown that BDNF is capable of stimulating the downstream PI3K/Akt signaling pathway via TrkB receptor activation, which in turn boosts the anti-apoptotic potential of neurons and diminishes secondary neuronal damage after cerebral hemorrhage (Hasegawa et al., 2020). Insufficient levels of BDNF result in decreased interactions among endothelial cells, potentially leading to ICH. An adequate supply of BDNF helps sustain endothelial cell stability, fosters cell-to-cell interactions, preserves the blood–brain barrier’s integrity, and consequently lowers the likelihood of bleeding (Donovan et al., 2000). BDNF is thought to be involved in the modulation of neuronal energy metabolism, which in turn boosts cellular resilience during ischemic or hemorrhagic occurrences. By augmenting the energy status within cells, BDNF allows neurons to more effectively endure different stress scenarios (Schäbitz et al., 2007). The external administration of BDNF could potentially enhance neurological recovery following a cerebral hemorrhage. Studies have demonstrated that BDNF administration results in notable neuroprotective effects and functional enhancements (Han et al., 2011; Lin et al., 2023).

Previous publications reported that the expression of TGF-β is significantly increased in the pathological state of ICH (Yang H. et al., 2019). According to Zhang et al., an increase in TGF-β following cerebral hemorrhage might play a role in the repair and regeneration processes of neurons and glial cells. TGF-β aids in the development of scar tissue and restricts the spread of the damaged region by enhancing glial cell proliferation and facilitating matrix remodeling (Zhang and Yang, 2020). Moreover, TGF-β is significant in modulating microglial activation and inflammatory responses, which are vital for managing neuroinflammation under pathological circumstances (Li Y. et al., 2017). TGF-β signaling can suppress heightened inflammatory responses, thereby safeguarding neurons. Additionally, TGF-β participates in the modulation of angiogenesis within the brain, which is crucial for the repair of injured tissue and enhancement of blood flow. It facilitates the development of new blood vessels by influencing the activities of endothelial cells (Zhang and Yang, 2020).

The possible involvement of IL-10 in neurological disorders is evidenced by its ability to inhibit neuroinflammation. During the recovery phase following intracerebral hemorrhage, the levels of IL-10 may further rise. This prolonged expression aids in both the resolution of inflammation and the restoration of injured tissue. Research indicates that the delivery of recombinant IL-10 enhances the long-term outcomes in mice deficient in IL-10, highlighting the crucial function of IL-10 in the recovery process (Han et al., 2023). Mice deficient in IL-10 demonstrate greater pathological injury following intracerebral hemorrhage, characterized by heightened brain edema and damage to white matter. In these mice, the phagocytic functions of macrophages and microglia were diminished, leading to a slower removal of hematoma, which in turn exacerbated tissue impairment (Li et al., 2021). Additionally, variations in IL-10 expression are significantly linked to other cytokines. For instance, the elevated levels of IL-17A following a stroke are associated with reduced IL-10, and the suppression of IL-17A may enhance the outcomes in mice lacking IL-10 (Piepke et al., 2021). This indicates that IL-10 not only contributes to its own anti-inflammatory properties but also interacts with other pro-inflammatory agents to collectively modulate the inflammatory response following cerebral hemorrhage.

### 5.4 Preclinical evidence of HPL therapy for ICH

**Figure 3:**
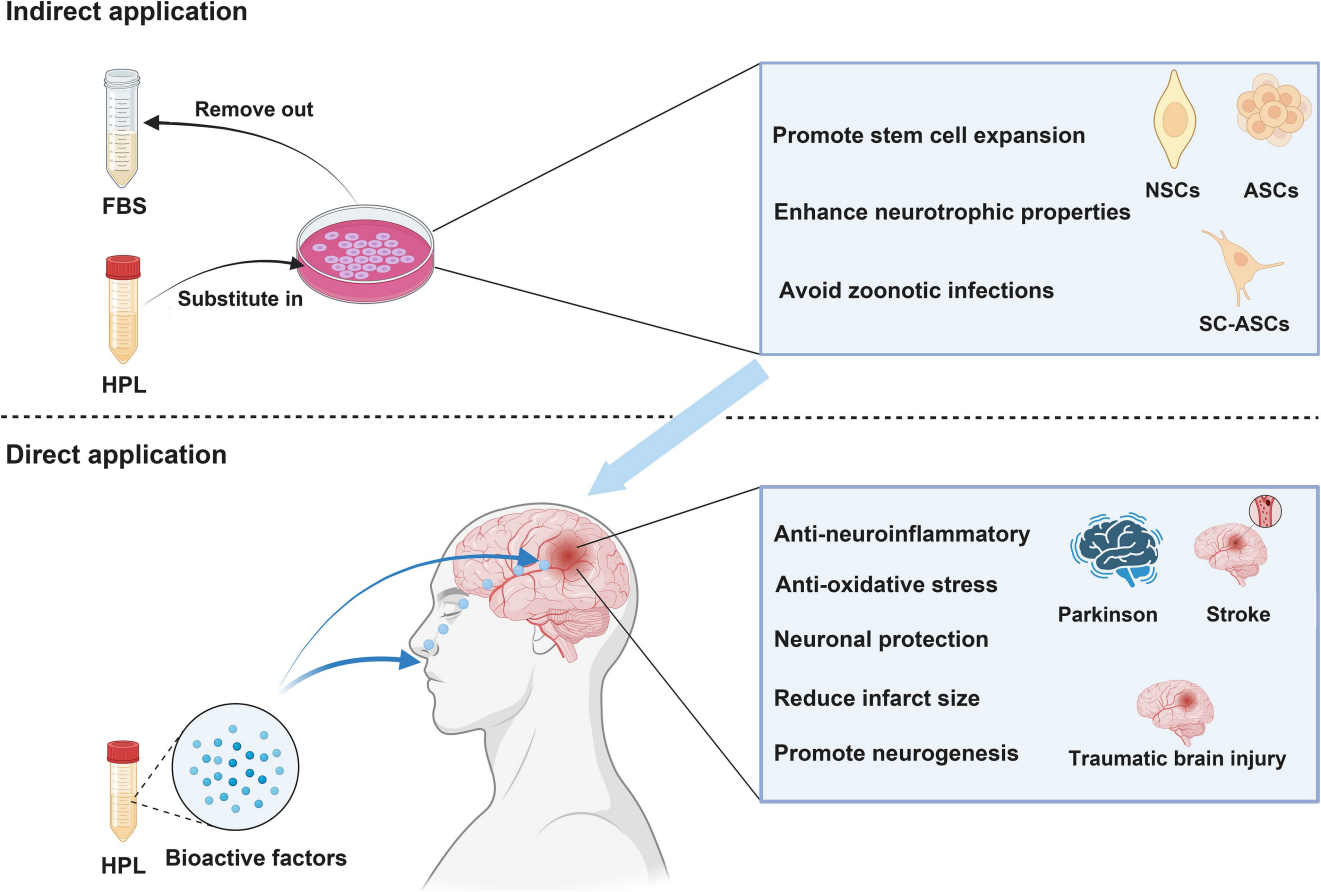
NSCs, neural stem cells; ASCs, adipose-derived stem cells; BMSCs, Bone marrow mesenchymal stromal cells. Indirect application: HPL increases the efficiency of cell therapy in treating neurological disease by culturing various types of stem cells. Direct application: HPL allows bioactive factors to directly reach the injured site and exert therapeutic effects through intranasal or targeted administration.

**Figure 4:**
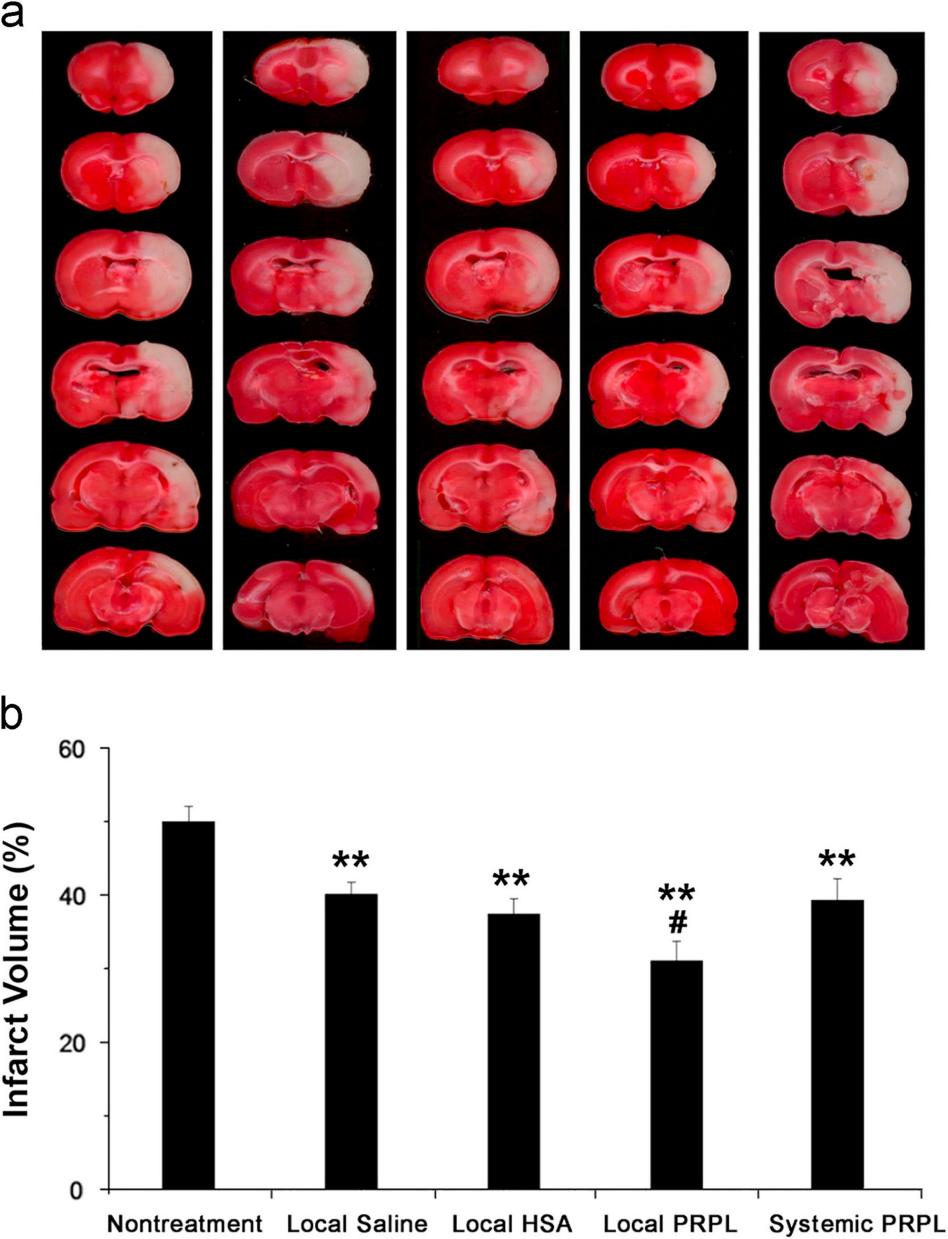
**(A)** Brain slices obtained from representatives stained with TTC 24 h post-reperfusion in cases of stroke, both with and without intervention. **(B)** Assessment of infarct volumes quantitatively across various groups shows that the infusion of local human PRP lysate markedly decreases infarct volume in comparison to the group with no treatment for stroke (**p < 0.01). Additionally, a further decrease is observed when this group is compared to the other treatment groups (^#^*p* < 0.05). The data is cited from a previous research (Zhang et al., 2015), this citation is permitted by the journal (License Number 5886510366832).

Furthermore, additional research has provided evidence that HPL directly influences areas related to neurological diseases for therapeutic purposes (Figure 3). An investigation into the intracerebroventricular delivery of HPL using a rat model characterized by middle cerebral artery occlusion marks the initial report validating the neuroprotective benefits of HPL in neurological disorders. Findings revealed that HPL was effectively administered to the lateral ventricle, facilitating angiogenesis, neurogenesis, and neuroprotection and leading to a decrease in behavioral impairments after cerebral ischemia (Hayon et al., 2013). Figure 4 shows the efficacy of HPL in mitigating ischemia–reperfusion injury in adult rats (Zhang et al., 2015). This research confirmed the neuroprotective properties of HPL, demonstrating its capability to decrease infarct volume and neurological impairments in experimental ischemic stroke. This effect may stem from the anti-apoptotic and anti-inflammatory properties of the growth factors present in HPL, as well as its potential to enhance vascular development and nerve regeneration. The benefits of HPL include: in contrast to individual exogenous neurotrophic or growth factors, the growth factors found in HPL are present in their natural forms and might exhibit synergistic effects. Furthermore, HPL possesses autologous qualities, minimizes the risk of infection or immune reactions, and is more cost-effective.

Nebie et al. employed HPL to deliver therapy to mice with traumatic brain injury (TBI) through intranasal administration. The group receiving treatment demonstrated enhancements in cognitive abilities and motor functions relative to the control group, alongside decreased levels of cortical neuroinflammation and oxidative stress (Nebie et al., 2021b). This research presents compelling support for HPL as a possible intervention for TBI. The investigators not only confirmed the functional efficacy of HPL in an *in vitro* setting, but they also assessed its therapeutic impact in two distinct *in vivo* systems, which enhanced the credibility of the findings. Additionally, the study indicated that HPL may operate through various mechanisms, such as enhancing neurotrophic factors, reducing inflammation, providing antioxidant protection, and facilitating neural repair. However, the dosages and methods of administration of HPL utilized in the experiments were derived from mouse studies, and these might not be directly relevant to human conditions. Furthermore, the investigation was solely reliant on animal studies and *in vitro* analyzes, lacking direct data from human clinical trials. Clinical trials involving humans are essential to confirm the safety and effectiveness of HPL. These aspects require further exploration during the translational medicine process.

*In vitro* studies have demonstrated that HPL provides a robust protective effect on neuronal cells subjected to ferroptosis inducers (Nebie et al., 2019). Additionally, in an Amyotrophic Lateral Sclerosis (ALS) mouse model, the administration of HPL through intracerebroventricular delivery and intranasal routes has been shown to extend the lifespan of the mice (Gouel et al., 2022). A 10-fold concentration of HPL, combined with heat treatment at 56°C, resulted in the diffusion of neurotrophic factors in the striatum following intranasal administration to mice with Parkinson’s disease, demonstrating neuroprotective effects (Chou et al., 2017). Anitua et al. evaluated the therapeutic effect of a specific human platelet lysate (HPL) enriched in growth factor plasma (PRGF-Endoret) in a mouse model of Alzheimer’s disease, The intranasal application of PRGF-Endoret in APP/PS1 mice showed a notable decrease in the accumulation of Aβ in the brain and a reduction in tau phosphorylation. Moreover, APP/PS1 mice treated with PRGF-Endoret demonstrated less astrocyte reactivity and a safeguarding effect against synaptic protein loss. *In vitro* experiments suggest that treatment with PRGF-Endoret influences astrocyte activation diminishes inflammatory responses, and enhances Aβ degradation. Furthermore, PRGF-Endoret administration resulted in significant improvements in behaviors related to anxiety, learning, and memory (Anitua et al., 2014). In the rat model of cerebral infarction, HPL has demonstrated significant therapeutic potential. Systemic infusion of HPL can reduce the size of cerebral infarction and enhance neurological scores in mice, particularly in the surrounding area of the lesion. Notably, local arterial administration exhibits a more pronounced therapeutic effect on cerebral infarction (Zhang et al., 2015). Additionally, HPL is utilized as a culture supplement for SH-SY5Y and BV-2 microglia cultures to assess cytotoxicity, evaluate inflammatory responses, and determine the capacity to stimulate wound healing, HPL modulates microglial activity while promoting both wound healing and neuronal differentiation (Nebie et al., 2021a). Another study evaluated the toxicity and neuroprotective activity of HPL on LUHMES cells and primary cortical/hippocampal neurons. The results indicated that HPL enhanced the expression of tyrosine hydroxylase and neuron-specific enolase in LUHMES cells, while it did not significantly affect synaptic protein expression in primary neurons. Furthermore, the tested virus-inactivated platelet lysates demonstrated potent neuroprotective effects on LUHMES and primary neurons exposed to erastin, a known inducer of ferroptotic cell death (Nebie et al., 2019). HPL demonstrates notable neuroprotective properties in cell-based models of Parkinson’s disease and amyotrophic lateral sclerosis, specifically in LUHMES and NSC-34 cell lines, respectively. It exhibits resistance to certain cell death pathways, including apoptosis and ferroptosis, as well as to specific oxidative stress inducers such as 1-methyl-4-phenylpyridine (MPP+) and menadione. The protective effects of HPL may be associated with the activation of the AKT pathway and the involvement of the MKE pathway (Gouel et al., 2017). A recent investigation thoroughly showcased the therapeutic capabilities of HPL in ICH, highlighting its role in decreasing neuroinflammation, mitigating oxidative stress, and offering neuronal protection (Qiu et al., 2024). Nevertheless, this research does not provide insights into the underlying mechanisms responsible for these effects. Consequently, future research avenues in clinical translation will concentrate on understanding the mechanisms by which these therapeutic effects manifest. More information is detailed in Table 3.

**Table 3:** HPL directly targets lesions to treat neurological diseases.

Model	Subject	Method of administration	Efficacy of HPL	References
TBI	Mice	Intranasal	Anti-neuroinflammatory, anti-oxidative stress, neuronal protection	Nebie et al. (2021b), Delila et al. (2023), Delila et al. (2024)
ALS	Primary motoneuron, Mice	*In vitro*, local injection, intranasal	Reducing iron deaths	Gouel et al. (2022)
Parkinson	Mice	Intranasal	Neuronal protection	Chou et al. (2017)
Stroke	Rat	local injection	Anti-neuroinflammatory, reducing infarct size	Hayon et al. (2013), Zhang et al. (2015)
Cell culture	SH-5YSY; BV-2 microglia	*In vitro*	Regulates microglial activation; promoting wound healing	Nebie et al. (2021a)

TBI, traumatic brain injury; ALS, Amyotrophic lateral sclerosis.

## 6 Synergistic effect of bioactive molecules in HPL in the treatment of ICH

### 6.1 Neuroinflammation

Neuroinflammation is frequently associated with both local and systemic damage. As inflammation propagates, it can initiate a positive feedback damage cascade. HPL contains numerous anti-inflammatory bioactive factors, including TIMP-1, IL-4, and miR-126-3p. Research has demonstrated that these factors exert synergistic effects on anti-neuroinflammation through various mechanisms (Wu et al., 2023). In recent years, the bioactive molecule tissue inhibitor of metalloproteinase TIMP-1 has been widely recognized for its inhibitory effect on inflammation. Furthermore, the imbalance between matrix metalloproteinases (MMPs) and TIMP-1 is thought to be associated with various neuroinflammatory diseases (Gardner and Ghorpade, 2003). Previous reports have indicated that neuroinflammatory diseases are associated with a reduction in TIMP-1 expression and an increase in MMP levels (Gardner et al., 2006). Current research suggests that TIMP-1 can inhibit MMP and exert anti-neuroinflammatory effects by binding to cell surface receptors (Thevenard et al., 2014; Knight et al., 2019; Nicaise et al., 2019). However, there are currently no studies reporting that HPL can increase the level of TIMP-1 in the body after ICH in some way. This is still a direction that we need to delve into in preclinical research. In addition, the bioactive molecule IL-4 in HPL is also an essential bioactive factor in reducing neuroinflammation (Nebie et al., 2022). A study examining the effects of IL-4 treatment on surgery-induced neuroinflammation in rats found that IL-4 significantly reduced microglial activation and the release of pro-inflammatory factors (Li Z. et al., 2017). In addition, the therapeutic effect of IL-4 on neuroinflammation was demonstrated in mice using exosomes containing IL-4 (Casella et al., 2018). IL-4 ameliorates neuroinflammatory diseases by interacting with IL-4 receptors in the nervous system (Vogelaar et al., 2018). The study conducted by Lima et al. also demonstrated the anti-neuroinflammatory effects of IL-4 in the context of spinal cord injury (Nebie et al., 2022). These studies demonstrate the broad therapeutic potential of IL-4 against neuroinflammation under different disease perspectives and different administration methods. There are also studies showing the anti-inflammatory effect of miR-126-3p in platelet exosomes and inhibiting the autophagy and death of cortical neurons by targeting the phosphoinositide-3-kinase regulatory subunit 2 (PIK3R2) in neonatal rats treated with hypoxia-reoxygenation injury (HI) (Ge et al., 2023).

### 6.2 Oxidative stress

Oxidative stress is primarily attributed to an increase in oxygen free radicals resulting from mitochondrial dysfunction (Ma et al., 2014) Other factors contributing to the production of reactive oxygen species (ROS) include the degradation of hemoglobin, which generates a substantial amount of ROS (Yang et al., 2017), and the infiltration of neutrophils, which also produces ROS (Zhao et al., 2017; Wu X. et al., 2024). Furthermore, studies indicate that inhibiting microglial activity can significantly reduce ROS levels (Wang and Tsirka, 2005; Yang et al., 2017; Zhao et al., 2017). Currently, there are no anti-oxidative stress drugs available for clinical use (Zheng et al., 2022), which is currently the biggest challenge to oxidative stress damage. Helmut Sies believes that oxidative stress is caused by the imbalance between oxygen free radicals and the body’s antioxidant capacity (Forman and Zhang, 2021). This indicates that HPL possesses the capability to reduce oxidative stress, as it contains a variety of antioxidant factors, including catalase (CAT), ceruloplasmin (CP), GPX, SOD, and GCLM (Nebie et al., 2021b). Research involving CP-deficient mouse models indicates that CP may mitigate oxidative stress by oxidizing non-iron substrates, including catecholamines and aromatic amines (Hineno et al., 2011). Folle discovered that GCLM can safeguard red blood cells by alleviating oxidative stress, which, in turn, diminishes the oxidative stress intensified by the degradation of red blood cells (Föller et al., 2013). Bhowmick et al. developed highly effective mimetics of GPx and Prx, which showed the ability to safeguard human cells against oxidative harm by displaying outstanding antioxidant properties in the presence of cellular thiols (Bhowmick et al., 2015). Studies have shown that administering exogenous superoxide dismutase (SOD) can lessen brain tissue injury caused by excitotoxic effects from sodium glutamate while also offering protection to mitochondria (Liao et al., 2020). Moreover, Research on Huntington’s disease has also indicated the antioxidant properties of GPX (Mason et al., 2013). The studies above provide compelling evidence that HPL effectively treats ICH by mitigating oxidative stress. Although these findings are primarily derived from preclinical research, the current absence of adequate clinical treatments for ICH underscores the necessity for further investigation into the potential of HPL as a therapeutic option. Moreover, several studies have shown that HPL is capable of activating the PI3K/AKT signaling pathway (Ranzato et al., 2009; Chadid et al., 2018). Interestingly, earlier research has proved that the activation of this pathway could reduce oxidative stress-related damage (Zheng et al., 2023; Wang J. B. et al., 2024).

### 6.3 Neuroapoptosis

Recent studies have highlighted the significant roles of Gelsolin, MANF, VEGF, and PDGF, bioactive factors that are abundant in HPL, in the protection of neuronal cells. Over the past few years, researchers have recognized at least 10 different mechanisms through which nerve cell death occurs. The pathways that result in the death of nerve cells are intricate and involve multiple factors. As a result, simply blocking a particular mechanism of nerve cell death might not be enough to avert cell loss (Fricker et al., 2018). Caspase, a protein specific to cysteinyl aspartate, is essential for the process of apoptosis and has an important function in neuronal cell death (Eskandari and Eaves, 2022). TNF-αattaches to death receptors on the cell membrane, resulting in the recruitment of Fas-associated death domain protein (FADD). This protein can interact with pro-caspase-8 to subsequently activate caspase-8, leading to the initiation of downstream signaling pathways that activate caspase-3 and ultimately induce apoptosis in the cell (Fricker et al., 2018). Studies suggest that a lack of neuron-specific caspase-8 results in diminished neuronal cell death, potentially linked to reduced activation of caspase-3 (Krajewska et al., 2011).

Gelsolin is a protein widely present in plasma and can also be detected in HPL (Zhang et al., 2011; Nebie et al., 2022). The study conducted by Zhang demonstrated that Gelsolin can decrease the activation of caspase-3, thereby mitigating neuroinflammation and neuronal death resulting from burns (Zhang et al., 2011).

MANF is a member of a newly identified family of neurotrophic factors (NTFs). In recent years, numerous studies have concentrated on its protective effects on nerve cells (Yang and Gao, 2020; Sivakumar and Krishnan, 2023). Studies have shown that applying rh-MANF significantly increased p-Akt, p-MDM2, and the Bcl/Bax ratios while simultaneously reducing the expression of p53, caspase-3, and neuronal death (Cao et al., 2023). Consequently, this intervention improved neurological function 24 h after ICH (Xu et al., 2018).

VEGF was identified as a target for tumor treatment following its discovery in the 1980s (Geindreau et al., 2021). However, its protective effect on nerve cells has been established by research conducted many years ago, Jin demonstrated that VEGF can inhibit the activation of caspase-3, thereby reducing the death of nerve cells induced by hypoxia (Jin et al., 2001). One year later, Svensson’s study demonstrated that VEGF can mitigate the excitotoxic neuronal cell death caused by exposure to methyl-d-aspartate (Svensson et al., 2002).

PDGF has been demonstrated to minimize MPP^+^-mediated neuronal cell toxicity, subsequently decreasing neuronal cell apoptosis, this protective effect may be achieved through the activation of the PI3K/AKT pathway (Chen H. et al., 2021).

### 6.4 Nerve regeneration and nerve damage repair

HPL contains a significant number of active factors that are related to the generation of neuronal cells, which is one of the primary reasons for its use as a therapeutic drug in cases of ICH. These factors that stimulate nerve growth are capable of enhancing the proliferation, migration, and differentiation processes of neural stem cells (NSCs) while also regulating the proliferation and development of Schwann cells. Moreover, they aid in axonal development and remyelination, contributing to neurogenesis within the hippocampus (Nebie et al., 2022). BDNF is found in substantial amounts in HPL. Recently, a growing body of research has indirectly underscored the essential function of BDNF in the development of nerves. The m-BDNF subtype binds to TrkB receptors, leading to the formation of phosphorylated TrkB receptors. This complex of m-BDNF and phosphorylated TrkB receptors activates various signaling pathways related to Rho family PI3K, MAPK, PLC-*γ*, and GTP-ases, thereby promoting neuronal dendrite growth, enhancing synaptic plasticity, and facilitating the growth of neuronal fibers (Kowiański et al., 2018). The impact of BDNF on promoting the growth of neural stem cells was validated through *in vitro* cultures of human embryonic spinal cord neural stem cells (NSCs) (Chen et al., 2013). EGF plays a vital role as a biological molecule that significantly aids in the regeneration of nerves. Recent studies have underscored the essential functions of EGF and its receptor (EGFR) in the processes of differentiation and regeneration of neurons and neural stem cells. EGF demonstrates considerable potential for promoting the neural differentiation of Dental Pulp Stem Cells (DPSC). This research expands on earlier studies that show DPSC’s ability to transform into neuronal precursors, thus providing new perspectives for treating neurological and neurodegenerative disorders (Lott et al., 2023). In addition, research has shown that the signaling pathway involving HB-EGF/EGFR could play a vital role in modulating the function of horizontal basal cells (HBC) and enhancing neurogenesis within the olfactory epithelium of zebrafish (Sireci et al., 2024). Notably, Li utilized the EGFR tyrosine kinase inhibitor PD168393 to suppress the EGFR signaling pathway, which allowed for an evaluation of the therapeutic impacts of PD168393 on spinal cord injury (spinal cord injury). The findings suggested that the EGFR inhibitor PD168393 significantly reduces excessive reactive astrogliosis and promotes a more favorable setting for axonal regeneration (Li et al., 2014). This research indicates that EGF might adversely affect SCI via its receptor, EGFR, but it also reveals that EGF facilitates astrogliosis. Significantly, a moderate degree of astrogliosis can benefit axonal regeneration (Liu and Neufeld, 2004; Sofroniew and Vinters, 2010; Norsted Gregory et al., 2013; Li et al., 2014). Research suggests that the EGF and its corresponding receptor EGFR are vital for facilitating neuronal differentiation and regeneration. In-depth investigations into EGF and its signaling pathways provide new perspectives and approaches for addressing nerve injuries.

Fibroblast growth factor (FGF) has been thoroughly examined regarding nerve regeneration following trauma. Due to the various biological regulatory processes linked to FGF, it has surfaced as a potential key player in facilitating the repair of injured neural tissue. The research conducted by Jungnickel offers an in-depth examination of the mechanisms that facilitate nerve regeneration after sciatic nerve damage in mice, achieved through the amplification of bFGF. Findings suggest that bFGF, produced naturally within the body, is essential in modulating the proliferation of Schwann cells, the regeneration of axons, and the process of myelination, which in turn affects the initial phases of peripheral nerve recovery (Jungnickel et al., 2006). This demonstrates the important role of FGF in the early neuroregeneration. A separate investigation indicated that FGF increases the movement ability of neural progenitor cells (NPCs), signifying its possible therapeutic importance in the ischemic cortex of neonates (Dayer et al., 2007). The impact of enhanced bFGF expression on axonal regeneration after nerve damage was studied through the use of a lentiviral vector (LV) to overexpress bFGF in the damaged peripheral nerves of rats. Findings revealed that the treatment with LV-bFGF promoted a faster reinnervation of the hindlimb muscles, particularly noted in those subjects that showed a greater count of motor and sensory neurons reaching the distal tibial nerve by the end of the observation period (Allodi et al., 2014).

By the close of the previous century, investigations into how insulin-like growth factor (IGF) influences neuronal regeneration intensified significantly. Research has shown that introducing IGF-1 into the peripheral bloodstream can promote the proliferation of neural progenitor cells (NPCs) in rats and specifically stimulate their differentiation into hippocampal neurons (Aberg et al., 2000). The study conducted by Breese suggests that IGF-1 may serve as a signal for local neuronal sprouting and reactive astrogliosis (Breese et al., 1996). In recent years, there has been a decline in preclinical evidence concerning the direct promotion of neuronal regeneration by IGF. Almengloinvestigated the potential of the N-terminal tripeptide of IGF-1, Gly-Pro-Glu (GPE), to promote nerve regeneration following injury. Their results support the hypothesis proposed by Almengló et al. (2017). Cochlear synaptic regeneration after excitotoxic trauma *in vitro* has been demonstrated to be promoted by IGF-1 (Yamahara et al., 2019).

Recent developments in exosome (EV) research have emphasized the promise of platelet-derived exosomes (PEV) within the domains of tissue engineering and biomedicine (Graça et al., 2023). Exosomes are tiny particles made up of lipid bilayers that are released by cells (Wang L. et al., 2024). This configuration has the ability to enclose proteins, RNA, and various other biologically active substances, and they are especially prevalent in HPL (Théry et al., 2018; Nebie et al., 2022). The distinctive physiological traits render it a hopeful option for therapeutic uses and drug transportation within regenerative medicine. Whether or not PEV directly facilitates nerve regeneration, it operates similarly to a vehicle that harnesses its physiological features to efficiently deliver neurotrophic and growth factors, including PDGF, BDNF, VEGF, and bFGF, due to its ability to traverse the blood–brain barrier (Ha et al., 2016; Rufino-Ramos et al., 2017; Agrahari et al., 2019).

### 6.5 Astrocytes and microglia

**Figure 5:**
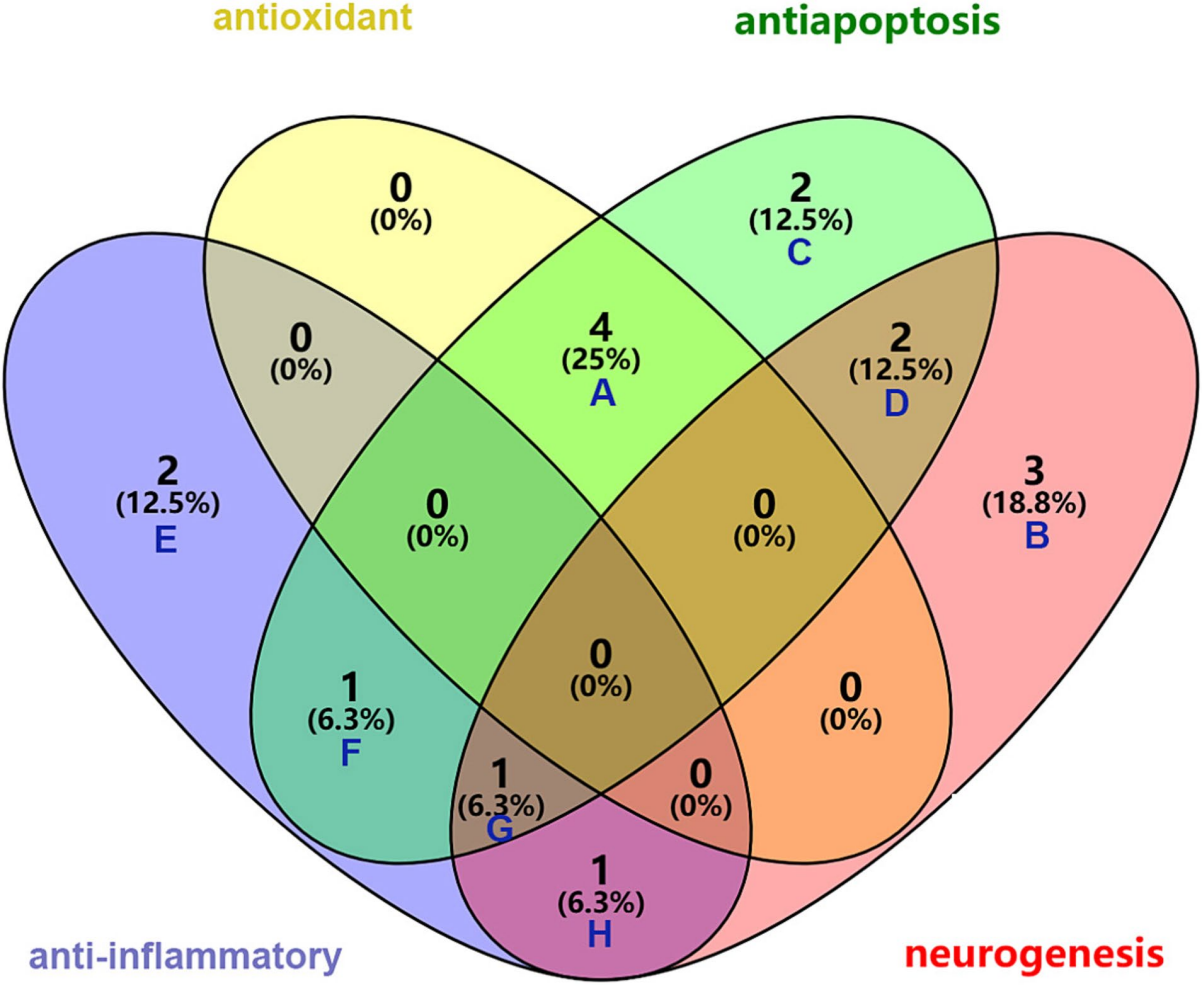
Synergistic role of bioactive molecules in HPL in the treatment of ICH. A: common elements in “antioxidant” and “antiapoptosis”: MANF, GPx, SOD, GAT; B: elements included exclusively in “neurogenesis”: EGF, TGF-β, PF4; C: elements included exclusively in “antiapoptosis”: GSN, MIR-126-3P; D: common elements in “antiapoptosis” and “neurogenesis”: PDGF, VEGF; E: elements included exclusively in “anti-inflammator”: FGF, IL-4; F: common element in “anti-inflammator” and “antiapoptosis”: IL-10; G: common element in “anti-inflammator,” “antiapoptosis” and “neurogenesis”: BNDF; H: common element in “anti-inflammator” and “neurogenesis”: IGF.

Astrocytes represent the most prevalent type of glial cells found in the central nervous system and are essential for the development of the BBB. Reactive astrocytes are considered markers of pathological states in the nervous system and are capable of responding to a diverse range of bioactive components present in HPL, such as FGF, EGF, TGF, and galectin-1 (LGALS1) (Linnerbauer et al., 2020; Sofroniew, 2020). Microglia serve as the innate immune cells that reside within the central nervous system (CNS). In the context of CNS inflammation, astrocytes alongside microglia are essential in managing this phenomenon by releasing a range of cytokines and inflammatory substances. Liddelow and colleagues showed that microglia in an activated state have the capacity to prompt astrocytes to take on a neurotoxic form. In particular, factors such as IL-1*α*, TNF-α, and complement component C1q released by microglia can activate astrocytes. These glial cells respond with specific transcriptional changes, which may result in the generation of neurotoxic substances, a decline in phagocytic function, and a decrease in the levels of neurotrophic factors (Liddelow et al., 2017). A separate investigation revealed that the signaling of AHR in microglia influences the expression of pro-inflammatory genes within astrocytes by affecting the levels of VEGF-B and TGF-α, VEGF-B facilitates the movement of NF-κB within astrocytes through FLT-1 signaling pathways, while TGF-α reduces the progression of experimental autoimmune encephalomyelitis (EAE) and stimulates the release of neuroprotective factors through ErbB1 receptor mechanisms (White et al., 2008; Rothhammer et al., 2018). In light of this, a potential approach for addressing neurological diseases could involve the suppression of reactive astrocyte and microglial activation. Studies indicate that galectin-1 helps alleviate neuroinflammation by deactivating M1 microglia via its interaction with sugar chains. Additionally, galectin-1 facilitates the maturation process of astrocytes while concurrently restraining their proliferation (Sasaki et al., 2004; Starossom et al., 2012). The intersection of the therapeutic effects of these bioactive molecules are illustrated in Figure 5 using a Venn diagram.

## 7 Challenges to be addressed and future prospects

Ongoing research efforts have led to some HPL studies entering the clinical trial stage targeting neurological disorders, which have raised significant safety concerns regarding its administration. However, its safety for brain applications requires additional validation through preclinical studies. Although the use of allogeneic PCs has emerged as a critical strategy for managing thrombocytopenic conditions, the advancement of HPL as a therapeutic option remains confined to the preclinical phase. Notably, allogeneic platelet biomaterials provide unique benefits compared with autologous transfusions. These benefits include the following: (1) autologous platelet derivatives often prove unsuitable for individuals with hematological disorders such as thrombocytopenia, platelet dysfunction, diabetes, or arteriovenous afflictions, and (2) autologous platelet derivatives can also be ineffective for specific chronic illnesses that require anticoagulant therapy, which may negatively affect the functional capacity of the patient’s own platelets. In the aging population, the incidence of such clinical situations is increasing, making it crucial to evaluate the safety of allogeneic treatments (He et al., 2022; Burnouf et al., 2023).

In addition, how to deliver the active ingredients in HPL to the diseased part is a major problem that must be solved. Since HPL is a blood-derived product enriched with bioactive agents, conventional methods of gastrointestinal and blood delivery are clearly inadequate. Local intracerebroventricular injection has been utilized in a few studies (Moreau et al., 2020); however, this method is invasive and may pose greater risks. Presently, most preclinical research employing HPL in animal studies indicates that intranasal delivery is the most effective method, and its effectiveness has been confirmed (Gouel et al., 2022; Le et al., 2024; Qiu et al., 2024). This technique enables the bypassing of the blood–brain barrier through the blood vessels and nerves found in the nasal cavity, promoting the efficient transport of HPL into the brain (Erdő et al., 2018). Based on these studies, it can be inferred that intranasal delivery, especially in the form of nasal spray or aerosolization, may play a crucial role in the clinical use of HPL.

HPL, as a blood-derived product, has a history of transmitting a range of diseases, such as HIV. Therefore, it is essential to create a framework of fair and effective regulations to guarantee that raw material sources are safe and that production methods are uncontaminated. This entails executing rigorous screening protocols for blood donors, testing for viruses in collected blood, and utilizing efficient techniques for virus inactivation and pathogen removal throughout the production process (Burnouf and Radosevich, 2000; Schallmoser et al., 2020). Moreover, ensuring consistency across the entire production process is crucial for guaranteeing the safety of the product (Bieback et al., 2019). Concerns regarding the safety of HPL are significantly heightened by viruses. Unlike bacteria and parasites, which can be efficiently eliminated using freeze–thaw cycles or bacterial filtration, viruses continue to represent a constant danger. To address the issue of virus safety in HPL, the International Society of Blood Transfusion stated that (Burnouf et al., 2019) HPL raw materials can only be sourced from licensed blood collection facilities, and the pool size of platelet lysate is restricted. In some institutions, the pool size of HPL is derived from 50 donors. The German Federal Regulatory Authority posits that the size of a pool should be restricted to a maximum of 16 individuals (Bieback et al., 2019). The presences of pathogens in HPL raw materials should be reduced by employing two photochemical virucidal technologies, namely INTERCEPT and Mirasol (Chen and Yao, 2016; Drew et al., 2017). Moreover, identifying pathogens in HPL collections and implementing specific virucidal treatments for extensive industrial batches are vital measures for ensuring safety. Furthermore, establishing traceability—developing a connection between blood donors, recipients, and resulting products—is critical for reducing risks and facilitating prompt actions (Burnouf et al., 2019).

Moreover, achieving consistency and standardization of production methods requires a global agreement. Efficient management and use of platelet products at blood supply centers play vital roles in preserving blood supply quality and ensuring patient safety. Each year, more than 100 million blood donations are gathered globally; however, only approximately 20% of PCs are deployed for standalone blood transfusions. Importantly, these raw materials can be redirected to creating HPL (Burnouf, 2019; Roberts et al., 2019). Owing to their limited shelf life, platelet products are more prone to expiration during both storage and distribution than red blood cells or plasma products. Differences in the ABO blood group system and HPL obtained from expired platelet plasma do not markedly affect the quality of the end product, but how blood centers handle expired platelet products is vital for improving the efficient use of blood resources and reducing waste (Jonsdottir-Buch et al., 2013; Strunk et al., 2018b).

Risks associated with HPL utilization are not validated. While numerous studies have indirectly highlighted the potential benefits of HPL in treating ICH, a majority of animal and cellular research has not documented the adverse effects related to HPL usage. Nonetheless, the absence of data from human clinical trials leaves unresolved potential hazards. HPL contains several elements that play a role in blood coagulation, which could foreseeably elevate the likelihood of thrombosis. It was observed in one study that hemostatic elements abundant in HPL might play a role in the likelihood of thrombosis induced by HPL (Wen et al., 2022). In individuals suffering from intracerebral hemorrhage, the presence of thrombosis can worsen their condition and potentially result in more severe complications. Due to the limited clinical studies available regarding HPL in the context of ICH, future investigations into this risk warrant careful consideration. Furthermore, personalized treatment should be prioritized. Prior to administering HPL, a thorough assessment of the patient’s history, including previous medical conditions and allergies, must be conducted to develop a tailored treatment plan. For those with a background of allergies or immune system disorders, careful deliberation is necessary regarding the use of HPL.

In summary, it is essential to further validate the safe and effective dosage of HPL through preclinical studies and clinical assessments, while also refining how these bioactive molecules and their signaling pathway modulators are administered. Growth factors not only enhance cell growth and proliferation but may also contribute to the progression of ICH. Thus, it is important to expand the focus beyond the beneficial effects of HPL to the context of disease. More research is necessary to elucidate its role in the onset of ICH, as well as to examine the interplay between its advantageous and adverse biological roles (Cognasse et al., 2021).

## 8 Conclusion

The advantages of HPL for using in culturing stem cells for cell therapy or directly targeting the nervous system are increasingly emerging. This mitigates the presumed risks associated with FBS in cell therapy and has the potential for large-scale production, as expired platelets can be utilized, thereby providing a cost advantage and reducing unnecessary waste. Consequently, HPL is a promising therapeutic avenue for the treatment of ICH. This review presents compelling evidence for the potential of HPL in the management of ICH. As research progresses, therapeutic strategies based on the biological activities of HPL are expected to provide new treatment options for ICH.
